# Urinary bladder obstruction caused by fungal balls: Unusual computed tomography findings – a case report

**DOI:** 10.1097/MD.0000000000048244

**Published:** 2026-04-10

**Authors:** Lan Zhang, Chengye Hong

**Affiliations:** a Department of Radiology, The Fourth Affiliated Hospital Zhejiang University School of Medicine, Yiwu, Zhejiang Province, PR China; b Department of Urology, The Fourth Affiliated Hospital Zhejiang University School of Medicine, Yiwu, Zhejiang Province, PR China.

**Keywords:** computed tomography, fungus ball, urinary bladder

## Abstract

**Rationale:**

Bladder fungal ball formation is a rare type of fungal infection, most commonly occurring in patients with diabetes or immunodeficiency. Only a few reports have described bladder fungal balls detected on computed tomography (CT). This case presentation highlights the characteristic CT findings of bladder fungal balls and discusses the diagnostic challenges.

**Patient concerns:**

A 66-year-old male was admitted with complains of passing particulate matter in urine, urinary frequency, urgency, and hematuria. He had a long-standing history of diabetes and hypertension.

**Diagnoses:**

Nonenhanced abdominal CT revealed multiple bladder nodules with mixed density, the largest measuring approximately 2.5 cm in diameter, accompanied by signs of emphysematous cystitis. The nodules exhibited a peculiar appearance. Pathological examination of expelled material and urine culture confirmed *Candida* species infection.

**Interventions:**

The patient received intravenous fluconazole for fungal urinary tract infection (400 mg daily) and intensive insulin therapy for critically high fasting blood glucose (23.31 mmol/L).

**Outcomes:**

After 10 days of treatment, follow-up CT showed a reduction of bladder gas shadows but enlargement of the nodules, filling the entire bladder lumen. Despite improved glycemic control, the patient refused further intervention (e.g., cystoscopic evacuation) and was lost to follow-up.

**Lessons:**

In high-risk patients (e.g., diabetes), the CT finding of multiple irregular bladder nodules with associated emphysematous cystitis should raise strong suspicion for bladder fungal balls, prompting timely urine culture and appropriate antifungal therapy.

## 1. Introduction

Fungal bladder infections are commonly observed in patients with long-term use of broad-spectrum antibiotics, diabetes, malignancies, or immunodeficiencies.^[1,2]^ Candida is a common pathogen responsible for fungal urinary tract infections.^[3,4]^ While candiduria is frequent, the formation of fungal ball (bezoars) consisting of intertwined hyphae, cellulose, and inflammatory cells within the bladder is rare. Although ultrasonography is often the initial imaging modality, computed tomography (CT) provides superior detail for characterizing lesion density, detecting intralesional gas, and excluding complications like fistulas. However, CT descriptions of bladder fungal balls remain scarce in the literature.^[5-9]^ We presented a case of a diabetic patient with multiple bladder fungal balls and emphysematous cystitis, detailing the distinctive CT features and discussing the diagnostic approach amidst limitations.

## 2. Case report

A 66-year-old male presented with a >1-year history of passing particulate matter in urine, which progressed over the past 2 months to include urinary frequency, urgency, and hematuria. His medical history included poorly controlled type 2 diabetes for over 10 years (on metformin and insulin) and hypertension for over 5 years (on nifedipine and irbesartan). Physical examination revealed no suprapubic tenderness. Digital rectal examination suggested benign prostatic enlargement. The International Prostate Symptom Score (IPSS) was 25 (severe symptoms).

**Figure 1.**
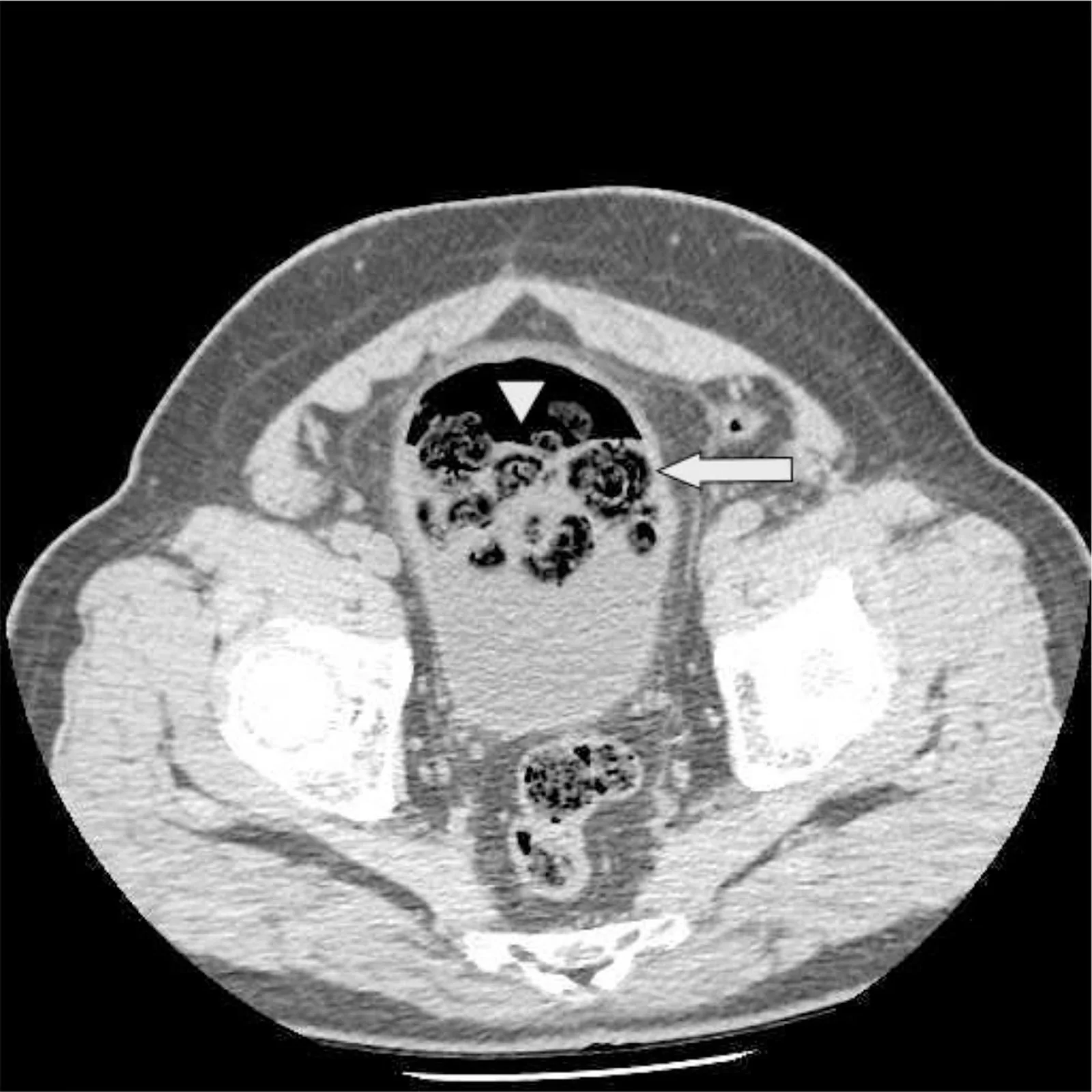
Axial noncontrast abdominal CT scan at admission shows multiple irregular, mixed-attenuation nodules (arrow) within the bladder lumen, associated with intraluminal gas (arrowhead), consistent with fungal balls and emphysematous cystitis.

Admission laboratory tests showed leukocytosis (white blood cell count: 11 × 10^9^/L) and elevated C-reactive protein (46.9 mg/L). Urinalysis showed turbidity, protein (3+), glucose (4+), numerous red blood cells (12,581/μL), and white blood cells (8098.6/μL). Blood glucose was critically elevated at 23.31 mmol/L. A noncontrast abdominal CT scan revealed multiple, well-defined, mixed-attenuation nodules filling the bladder lumen, the largest measuring approximately 2.5 cm in diameter (average attenuation −170 Hounsfield Units). The nodules had irregular surfaces, with some showing small gas bubbles. Significant intraluminal gas with air-fluid levels was noted, consistent with emphysematous cystitis (Fig. 1). The bladder wall appeared diffusely thickened, but no definite fistulous tract to the bowel was identified. The patient passed several soft, friable masses per urethra.

**Figure 2.**
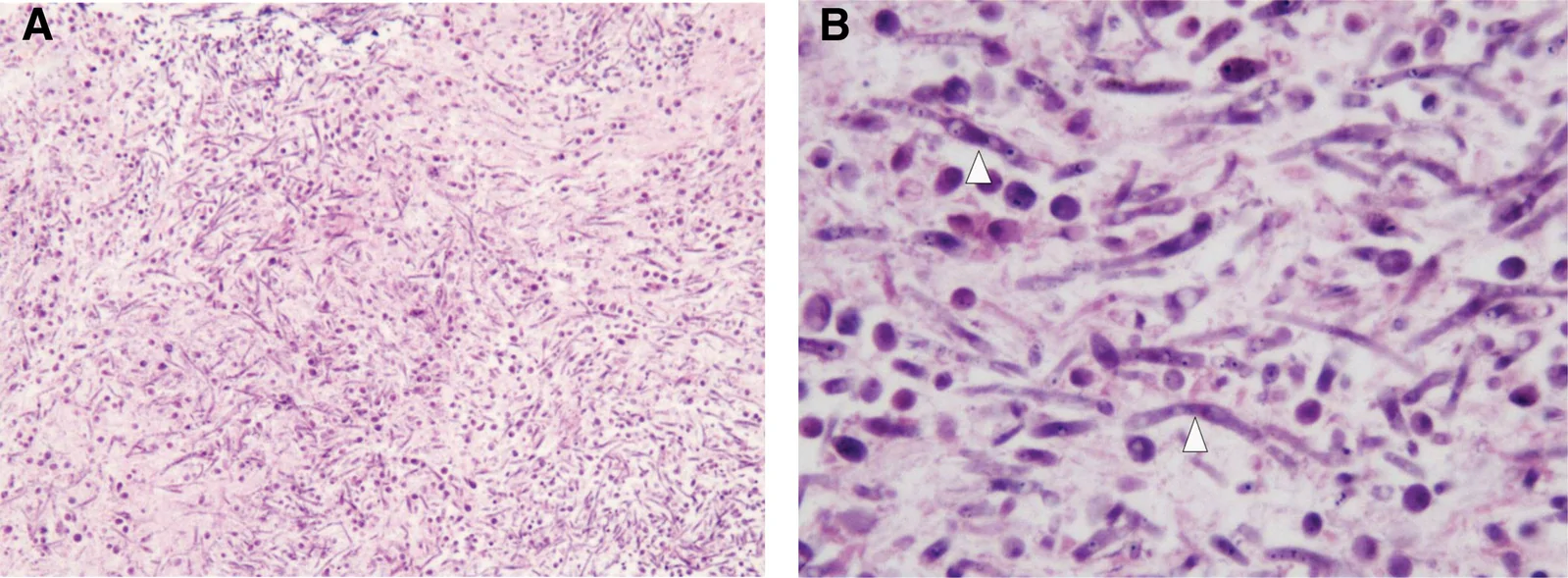
Photomicrographs of the expelled fungal ball (H&E stain). (A) Low-power view (×100) showing dense infiltration of inflammatory cells (lymphocytes and plasma cells) along with numerous fungal hyphae. (B) High-power view (×400) demonstrating numerous fungal hyphae with a characteristic moniliform appearance (arrowhead), a morphologic hallmark of Candida species.

Pathological examination of the expelled masses (hematoxylin and eosin staining) revealed dense infiltrates of inflammatory cells (including lymphocytes and plasma cells) and numerous fungal hyphae at low magnification (×100; Fig. 2A). At higher magnification (×400), the fungal hyphae exhibited a characteristic moniliform appearance, which is a morphologic hallmark of Candida species (Fig. 2B). Urine culture subsequently confirmed *Candida tropicalis*.

**Figure 3.**
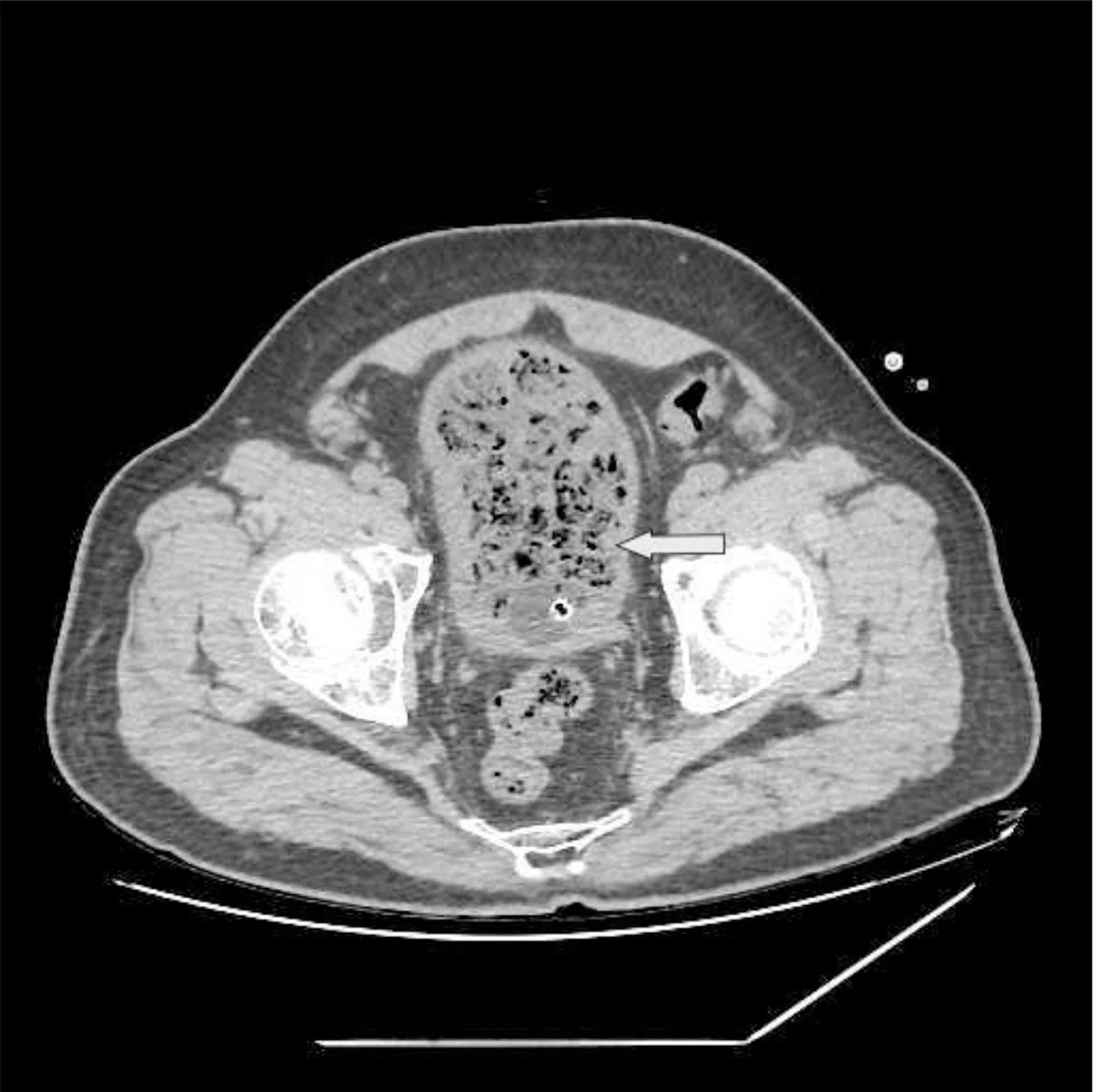
Follow-up axial noncontrast CT scan after 10 days of antifungal therapy demonstrates near-complete filling of the bladder lumen by enlarged, coalescing nodules (arrow), with resolution of the previously seen gas.

He was treated with intensive insulin therapy and intravenous fluconazole (400 mg daily). This initial medical management aimed to: stabilize the patient’s severe hyperglycemia to reduce infection risk and optimize his condition for any potential future procedure; assess the early response to systemic antifungal therapy in a patient who was hesitant about invasive interventions. After 10 days, a repeat CT was performed to evaluate treatment response. It showed resolution of the intramural gas but a marked increase in the size and number of the nodules, now causing near-complete bladder outlet obstruction (Fig. 3), indicating inadequate response to medical therapy alone. Despite clear explanation of the necessity for procedural intervention to relieve the obstruction, the patient declined further invasive treatment for personal reasons. He acknowledged the risks and signed an informed refusal form before discharge.

## 3. Discussion

The immune system impairment and high tissue glucose levels in patients with diabetes can create a favorable environment for microbial growth and proliferation.^[2]^ In this case, the urinary tract fungal infection was likely related to the patient’s long-standing diabetes and poor glycemic control.

The clinical manifestations of fungal bladder infections are often nonspecific, ranging from asymptomatic to mild symptoms, making them easy to overlook.^[3]^ In our case, the patient initially presented with urinary abnormalities, which progressed over a year to symptoms of urinary irritation such as frequency, urgency, hematuria, abdominal pain, and pneumaturia (possibly due to Candida fermenting urinary glucose). This suggests an asymptomatic period, during which the infection gradually progressed, eventually leading to necrotic tissue detachment and fungal ball formation.

The CT findings in our case are noteworthy. The nodules showed mixed attenuation, likely reflecting the complex composition of fungal elements. This appearance, however, can mimic an enterovesical fistula due to the presence of intraluminal gas and debris. Key differentiating points include the absence of a discernible fistulous tract, lack of extraluminal inflammation connecting to bowel, and clinical absence of fecaluria. Other differentials include blood clots or rare tumors, but these typically do not associate with intraluminal gas.

A review of recent case reports reveals varied presentations of bladder fungal balls.^[5-9]^ While some describe solitary lesions, others, like ours, report multiple nodules causing significant obstruction.^[5-7]^ The most similar documented CT appearances include multiple uniform nodules resembling foreign bodies and nodules intermixed with air bubbles.^[8,9]^ The mean CT attenuation of the nodules in our case was −170 HU, substantially lower than the value of 96.6 HU reported by Baten et al, indicating a higher gas content within the fungal mass in our patient.^[9]^ This highlights the variable imaging spectrum, but the combination of irregular, mixed-attenuation filling defects with gas in the bladder lumen, especially in a high-risk host, remains highly suggestive.

The management of bladder fungal balls typically involves a combination of systemic antifungal therapy and procedural intervention to relieve obstruction.^[3,6]^ Medical therapy alone (e.g., with fluconazole) is often insufficient to clear large fungal bezoars, as demonstrated in our case, but is crucial for controlling systemic infection and as an adjunct to procedures.^[6]^ Procedural options include cystoscopic evacuation or dissolution with irrigation, which is minimally invasive and effective for removing the obstructing material.^[6]^ The choice depends on the size and number of fungal balls, the patient’s overall condition, and local expertise. In our patient, the initial medical approach was chosen due to severe hyperglycemia and patient reluctance, but the progression of obstruction underscored the eventual need for mechanical evacuation, which the patient ultimately refused.

The primary limitations of this report are the lack of long-term follow-up and definitive procedural intervention, which precludes confirmation of resolution and limits evaluation of optimal treatment efficacy. This also highlights an ethical and practical challenge when patients decline standard care. Nevertheless, the distinctive CT and corresponding pathological findings provide valuable educational insight into this rare condition.

## 4. Conclusions

Bladder fungal balls, though rare, should be considered in the differential diagnosis of filling defects in the bladder, especially in diabetic or immunocompromised patients with unexplained emphysematous cystitis. CT can reveal characteristic features, such as irregular, mixed-attenuation nodules sometimes containing gas, which can guide diagnosis when combined with urine culture. Early diagnosis and a combination of medical and procedural interventions are crucial for successful outcomes, though patient compliance remains a critical factor.

## Author contributions

**Conceptualization:** Lan Zhang, Chengye Hong.

**Data curation:** Lan Zhang, Chengye Hong.

**Investigation:** Lan Zhang, Chengye Hong.

**Resources:** Lan Zhang, Chengye Hong.

**Supervision:** Lan Zhang.

**Validation:** Lan Zhang, Chengye Hong.

**Writing – original draft:** Lan Zhang, Chengye Hong.

**Writing – review & editing:** Lan Zhang, Chengye Hong.
